# An optimization method for flexible interconnection planning based on improved CNN-LSTM prediction and tunable relative entropy-driven chaotic evolution

**DOI:** 10.1371/journal.pone.0351324

**Published:** 2026-06-12

**Authors:** Xiaoyan Zhao, Xubin Xing, Xiaoyan Guo, Jian Chao, Dapeng Hu, Xiangtao Zhuan

**Affiliations:** 1 Zhuhai Power Supply Bureau of Guangdong Power Grid Co., Ltd., Zhuhai, Guangdong, China; 2 School of Electrical Engineering and Automation, Wuhan University, Wuhan, Hubei, China; University of the Philippines Diliman, PHILIPPINES

## Abstract

As power systems evolve towards greater intelligence and flexibility, flexible interconnection technology has emerged as a critical means to enhance operational reliability and economic performance. This paper presents a data-model dual-driven planning methodology for flexible interconnection systems, integrating a multi-scale spatio-temporal cross-enhanced CNN-LSTM model for load forecasting with a Chaotic Evolutionary Optimization (CEO) algorithm to optimize system design. The proposed framework first constructs an improved CNN-LSTM hybrid architecture, trained on historical load data and simulated feature sets, to predict future load profiles. A novel Tunable Relative Entropy (TRE) metric is introduced as a complementarity quantification index, forming a multi-objective function that incorporates system balance, reliability, economy, and spatio-temporal complementarity. The CEO algorithm is then employed to solve the optimization model, determining the optimal system configuration and operational parameters. Experimental evaluations demonstrate that the forecasting module achieves high accuracy, with a Mean Squared Error (MSE) of 0.000368 and a Mean Absolute Error (MAE) of 0.006334. Moreover, the TRE index improves complementarity efficiency by 3.8%. By leveraging the predictive capability of the hybrid neural network and the CEO algorithm’s optimization efficacy, the proposed approach not only reduces load fluctuation indices but also enhances planning efficiency and operational economy, offering a viable pathway for intelligent power system development.

## 1 Introduction

Against the backdrop of the global energy transition and power market reform, modern power systems face multi-faceted challenges, including heightened load volatility and increasing penetration of renewable energy sources [1,2]. As a key solution to these issues, flexible interconnection technologies enable spatio-temporal optimization of power resource allocation by establishing multi-regional power interaction networks [3–5]. However, the planning and operation optimization of these devices involve high-dimensional, nonlinear decision-making problems, where conventional methods often exhibit limitations in solving efficiency and global optimization capability [6].

Load forecasting, as a fundamental link in the planning of flexible interconnection systems, directly affects the subsequent optimization effect. The application of traditional forecasting techniques, such as time series analysis and Kalman filtering, is constrained by inherent deficiencies in addressing nonlinearities and high-dimensionality within datasets, thus failing to adequately capture the differentiated patterns inherent to regional loads [7]. In recent years, deep learning technology has accounted for an increasing proportion in the field of time series forecasting, such as Convolutional Neural Network (CNN); however, deep learning methods generally have problems such as difficulty in determining internal parameters and slow calculation speed [8]. In response to this, hybrid forecasting methods have emerged, combining the characteristics of different types of neural networks to improve the overall optimization performance [9–14].

In recent years, further research on deep learning technology has improved forecasting performance [10]. The CNN-LSTM hybrid model combines the spatial feature extraction capability of CNN and the time series modeling capability of LSTM, and has achieved remarkable results in power load forecasting. For example, Reference [15] proposes using a hybrid CNN and GRU neural network for load forecasting, which helps to reduce waste and address issues related to demand-side management. In addition, there are load forecasting methods that combine traditional forecasting methods with neural networks; Reference [16] employs stacking to integrate base models such as ANN, XGBoost, and LSTM, and uses Lasso regression as the meta-model to fuse the prediction results. Due to the uncertainty of load forecasting itself, the forecasting is affected by various factors; Reference [17] combines feature engineering with Fourier transform and wavelet transform, and through enriching the data and optimizing the model performance, significantly improves the accuracy of power load forecasting. Furthermore, Reference [18] integrates empirical mode decomposition into the neural network model, and combines the Bayesian optimization strategy. The feature decomposition is used to enhance the accuracy of the model.

In addition to load forecasting, the scheduling of the power grid itself is also extremely important. However, research on the installation and scheduling of flexible interconnection devices is still not comprehensive [19–21]. In terms of the overall optimization of flexible interconnection systems, the objective function of flexible interconnection usually involves multi-objective optimization problems such as balance, reliability, and economy [22–24]. Reference [25] conducts adaptive scheduling for flexible interconnection stations in multi-level operating states and proposes an adaptive weight coefficient adjustment method. The work in Reference [26] focuses on flexible interconnection planning within low-voltage distribution networks to improve power supply capacity. However, conventional mathematical programming techniques, including linear and dynamic programming, often face challenges related to prohibitive computational complexity and inadequate global exploration, especially in large-scale or non-convex problems. Consequently, heuristic algorithms have gained prominence as a prominent alternative, favored for their flexibility and robustness in navigating complex, constrained search spaces.

Recent studies have further advanced interconnection planning. For instance, robust planning methods for hybrid microgrids have been proposed to fortify systems against renewable uncertainty [27], and risk-based data-driven approaches have been applied to integrated energy management [28], highlighting the trend towards intelligent optimization.

The optimization of flexible interconnection systems involves equipment configuration, operation scheduling, energy management, and other aspects. Traditional studies mostly adopt mathematical programming methods, such as Mixed Integer Linear Programming (MILP) and Nonlinear Programming (NLP); however, these methods have high computational costs when dealing with large-scale complex systems. Heuristic algorithms such as Genetic Algorithm (GA), Particle Swarm Optimization (PSO), and Ant Colony Optimization (ACO) have become important tools for power system optimization due to their global search capabilities and adaptability to nonlinear problems. For example, Reference [29] suggests using heuristic algorithms to optimize the configuration of flexible interconnections. Based on this research, this paper adopts the Chaotic Evolutionary Optimization (CEO) algorithm combined with chaotic evolution theory, which has high exploration capability and convergence speed, making it suitable for solving such problems [30].

Based on the described context, this paper proposes a data-model dual-driven planning method for flexible interconnection systems. The approach employs an improved CNN-LSTM model to achieve high-precision load forecasting, and utilizes the CEO algorithm to optimize system configuration and operational strategies. This integrated methodology enhances both the planning efficiency and economic performance of flexible interconnection systems. The main innovations of this work are as follows: (1) an enhanced CNN-LSTM hybrid model is designed, incorporating multi-scale convolution, bidirectional LSTM, and an attention mechanism to improve load forecasting accuracy; (2) a novel Tunable Relative Entropy (TRE) index is introduced as a measure of complementarity. A multi-objective function is constructed that simultaneously considers balance, reliability, economy, and spatio-temporal complementarity. Efficient optimization is achieved through a chaotic optimization algorithm.

## 2 Methodology

### 2.1 Improved CNN-LSTM load forecasting model

The proposed CNN-LSTM architecture introduces three critical enhancements over baseline models: (1) Multi-scale 1D convolutions (kernels 3 and 5) for hierarchical spatial feature extraction; (2) Bidirectional LSTM with residual connections to mitigate vanishing gradients and capture long-term dependencies; (3) 8-head attention mechanism focused on peak-load time points. These modifications specifically address the spatio-temporal complexity of regional load patterns.

**Fig 1:**
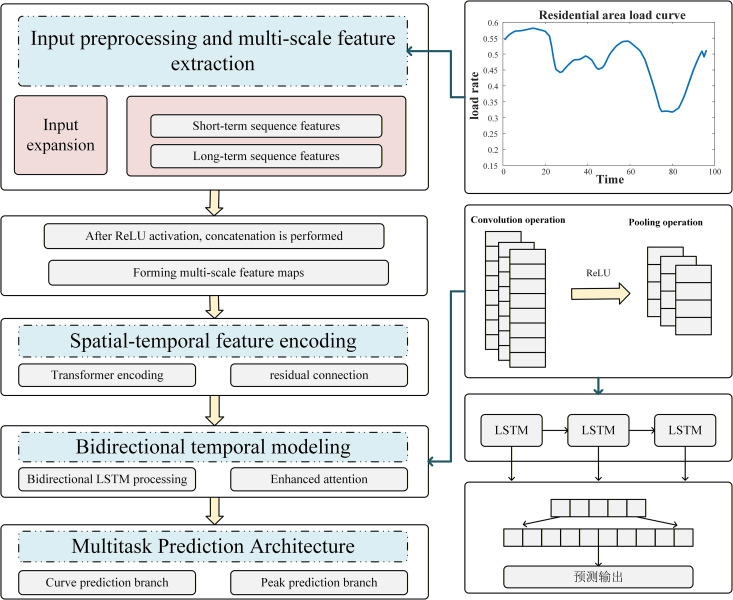
Flowchart of the hybrid neural network process.

To achieve high-precision load forecasting, this paper designs an improved CNN-LSTM hybrid model, which introduces an attention mechanism and multi-task branch forecasting on the basis of the original hybrid model [31]. Its structure is shown in Fig 1. First, it receives normalized regional feature data, including area, population, electricity consumption type, and climate characteristics. The input matrix is:


$$\begin{array}{c} 𝐙=[𝐑,𝐁,𝐈,d_{\text{model}}] \end{array}$$ (1)

where **Z** is the input matrix, **R** represents the input features after linear transformation introduced through residual connection, **B** is the batch size, **I** is the sequence length, and *d*_model_ denotes the model dimension.

In the multi-scale CNN module, short-kernel and long-kernel convolutions are used to extract spatial features of different scales, and nonlinear transformation is performed through the ReLU activation function:


$$\begin{array}{c} s_{1}=\text{ReLU}(\text{Conv1d}(𝐱,64,3,\text{padding}=1)) \end{array}$$ (2)

$$\begin{array}{c} s_{2}=\text{ReLU}(\text{Conv1d}(𝐱,64,5,\text{padding}=2)) \end{array}$$ (3)

$$\begin{array}{c} 𝐜={\text{ReLU}}_{\text{concat}}(s_{2},l_{2}) \end{array}$$ (4)

where *s*_1_ is the short-convolution path, *l*_2_ is the long-convolution path, Conv1d(·,C,K,padding=P) represents the one-dimensional convolution operation with the number of output channels *C*, convolution kernel size *K*, and padding *P*, and **c** is the concatenated feature tensor.

To retain the original input information and alleviate the gradient vanishing problem, residual connection is introduced. First, the input dimension is adjusted through a linear layer to expand **r** into ${𝐫}^{\prime }$ and add it to the Transformer output. A bi-directional LSTM module is adopted to capture the long-term dependence of the load curve and improve the time series modeling effect. Among them, Bi-LSTM is the bi-directional LSTM network, and its output is ${\text{lstm}}_{\text{out}}=[𝐁,𝐃,512]$, where 512 is the dimension after concatenation of forward and backward hidden states. An 8-head attention mechanism is used to enhance the focus on key time points, followed by multi-head segmentation:


$$\begin{array}{c} Q_{h}=Q.\text{view}(𝐁,-1,H,D_{h}).\text{transpose}(1,2) \end{array}$$ (5)

$$\begin{array}{c} K_{h}=K.\text{view}(𝐁,-1,H,D_{h}).\text{transpose}(1,2) \end{array}$$ (6)

$$\begin{array}{c} V_{h}=V.\text{view}(𝐁,-1,H,D_{h}).\text{transpose}(1,2) \end{array}$$ (7)

where h∈{1,…,H} indexes the attention head, *H* is the number of attention heads, and $D_{h}=512/H$ is the dimension per head. First, linear transformation is performed to generate query *Q*, key *K*, and value *V*. Adaptive average pooling is used to compress the features into a fixed-size vector. Then, the fully connected layer outputs the load curve prediction values and peak prediction at 96 time points:


$$\begin{array}{c} y_{\text{curve}}={\text{Linear}}_{\text{curve}}(p) \end{array}$$ (8)

$$\begin{array}{c} y_{\text{peak}}={\text{Linear}}_{\text{peak}}(p) \end{array}$$ (9)

where *p* denotes the feature vector after adaptive average pooling.

The model training adopts the adamw optimizer (learning rate = 0.001, weight decay = 10^–5^) and cosine annealing learning rate scheduler. The loss function is Mean Squared Error (mse), supplemented by the auxiliary loss of peak prediction.

### 2.2 Chaotic evolutionary optimization (CEO) algorithm

**Fig 2:**
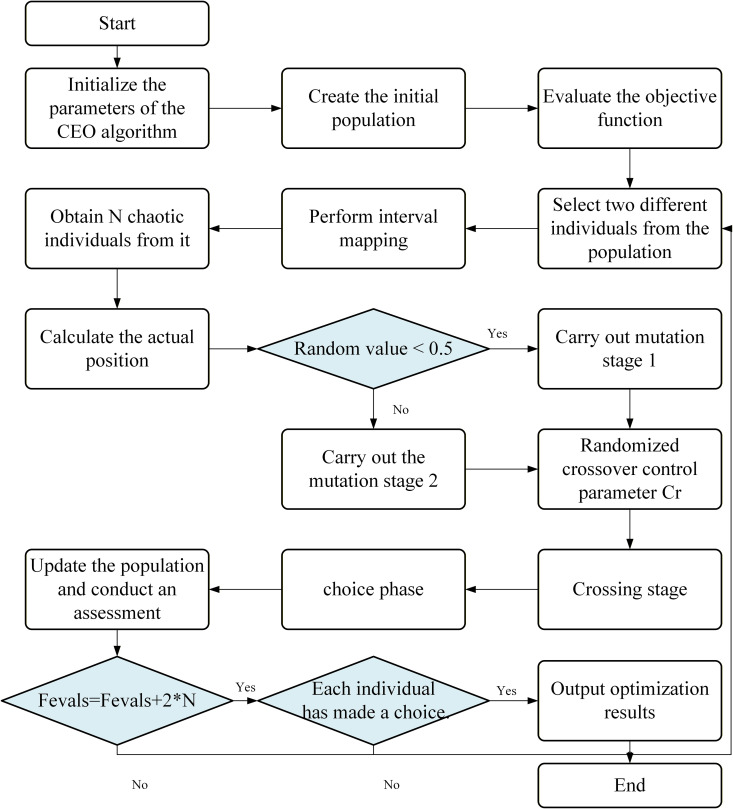
Flowchart of the chaotic evolutionary algorithm.

The algorithm flow consists of the following steps: initialization of the population, calculation of fitness using the objective function Cost, update of positions, and iteration until convergence is achieved, culminating in the output of the optimal configuration parameters **X** and power allocation *P*_*n*_. The overall algorithm is shown in Fig 2.

Population initialized via Latin Hypercube Sampling within [*lb*, *ub*] bounds; *iter* = current iteration index; *n* = individual index in population ($n=1,\ldots ,N_{\text{pop}}$), Best_*t*_ = individual with minimal Cost in current population (applies to both **x** and **y** trajectories); the convergence criterion is $|{\text{Cost}}_{iter}-{\text{Cost}}_{iter-1}|<10^{-4}$ or max iterations reached.

The overall CEO algorithm is a population-based evolutionary algorithm, and its unified search framework is first given as:


$$\begin{array}{c} x_{iter+1}=x_{iter}+\alpha \cdot d_{iter} \end{array}$$ (10)

where *x*_*iter*_ and *x*_*iter+1*_ are the current individual and mutated individual, respectively; α represents the search step size; and *d*_*iter*_ is the evolutionary direction.

The CEO algorithm adopts the hyperchaotic characteristics of the two-dimensional heterogeneous group hyperchaotic map, where two individuals *x*_*iter*_ and *y*_*iter*_ must be mapped to the range of the following equations respectively:


$$\begin{array}{c} \left\{ \begin{array}{c} x_{iter}^{\prime }=\frac{x_{iter}-lb}{ub-lb}-0.5\\ y_{iter}^{{\;}^{\prime }}=0.5\frac{y_{iter}-lb}{ub-lb}-0.25 \end{array}\right. \end{array}$$ (11)

where *lb* and *ub* are the lower and upper bounds of the population variables, respectively. Then, inverse mapping is performed on them:


$$\begin{array}{c} \left\{ \begin{array}{c} x_{\text{chaos}}^{n^{\prime }}=(x_{\text{chaos}}^{n}+0.5)(ub-lb)+lb\\ y_{\text{chaos}}^{n^{\prime }}=2(y_{\text{chaos}}^{n}+0.25)(ub-lb)+lb \end{array}\right. \end{array}$$ (12)

Multiple evolutionary directions are generated according to the mapped individuals:


$$\begin{array}{c} \left\{ \begin{array}{c} d_{x,iter}^{n}=x_{\text{chaos}}^{n^{\prime }}-x_{iter}\\ d_{y,iter}^{n}=y_{.}chaos^{n^{\prime }}-y_{iter} \end{array}\right. \end{array}$$ (13)

where n∈{1,2,…,N}, and $d_{x,iter}^{n}$ and $d_{y,iter}^{n}$ are the evolutionary directions of the corresponding individuals, respectively.


$$\begin{array}{c} \left\{ \begin{array}{c} {\tilde{x}}_{iter+l}^{n}=x_{iter}+a(x_{\text{chaos}}^{n^{\prime }}-x_{iter})\\ {\tilde{y}}_{iter+l}^{n}=y_{iter}+a(y_{\text{chaos}}^{n^{\prime }}-y_{iter}) \end{array}\right. \end{array}$$ (14)

where ${\tilde{x}}_{iter+1}^{n}$, ${\tilde{y}}_{iter+1}^{n}$ are the positions of individuals mutated by chaotic mapping, and *a* is the search step size of the algorithm, which acts on the mutated individuals generated from the original individuals. To further improve the local development capability of the algorithm, the search for nearby optimal solutions is continued to accelerate the convergence speed of the algorithm:


$$\begin{array}{c} \left\{ \begin{array}{c} {\tilde{x}}_{iter+1}^{n}=Best_{iter}+a(x_{\text{chaos}}^{n^{\prime }}-x_{iter})\\ {\tilde{y}}_{iter+1}^{n}=Best_{iter}+a(y_{\text{chaos}}^{n^{\prime }}-y_{iter}) \end{array}\right. \end{array}$$ (15)

where Best_*iter*_ is the optimal solution of the current population.

### 2.3 Tunable relative entropy (TRE) for complementarity quantification

To enhance the coherence of the index system in flexible interconnection planning, it is proposed to introduce Tunable Relative Entropy (TRE) as a complementarity quantification index, which dynamically measures the spatio-temporal complementarity of load curves between feeders from the perspective of probability distribution.

For the load curves *P*_*a*_(*t*_*k*_) and *P*_*b*_(*t*_*k*_) of two feeders, the time-varying relative entropy is defined as:


$$\begin{array}{c} D(P_{a}||P_{b},t_{k})=\sum_{t_{k}}P_{a}(t_{k})\log\left(\frac{P_{a}(t_{k})+\varepsilon }{P_{b}(t_{k})+\varepsilon }\right) \end{array}$$ (16)

where ε is a smoothing constant (to avoid division by zero). A tunable parameter α∈[0,1] is introduced to construct a bidirectional complementarity measure:


$$\begin{array}{c} C_{\text{comp}}(t_{k})=\alpha \cdot D(P_{a}||P_{b},t_{k})+(1-\alpha )\cdot D(P_{b}||P_{a},t_{k}) \end{array}$$ (17)

where a larger *C*_comp_(*t*_*k*_) indicates a more obvious negative difference between the two feeders at time *t*_*k*_.

Aggregation in the time dimension is performed to generate a normalized evaluation index; a value closer to 1 indicates strong complementarity in the full time period:


$$\begin{array}{c} C_{\text{norm,comp}}=\frac{1}{K}\sum_{k=1}^{K}\frac{C_{\text{comp}}(t_{k})}{\max(C_{\text{comp}}(t_{k}))} \end{array}$$ (18)

where *C*_*norm*,*comp*_ is the normalized complementarity assessment index, and the closer its value is to 1, the stronger the all-time spatio-temporal complementarity between feeders is, *k* indexes the *K* discrete time intervals.

## 3 Establishment of the overall model

### 3.1 Flexible interconnection model

In this study, the performance indicators of flexible interconnection are divided into three parts, fully considering the role and application requirements of flexible interconnection devices. This subsection defines three normalized performance indicators for flexible interconnection evaluation: (1) Load balance degree, (2) Device utilization, (3) Mutual aid balance strength. The indicators are summarized as follows:

#### 3.1.1 Daily average balance degree of interconnected feeders (normalized).


$$\begin{array}{c} C_{\text{bal}}=\frac{1}{nK}\sum_{i=1}^{n}\sum_{k=1}^{K}\alpha_{i}(t_{k}) \end{array}$$ (19)

$$\begin{array}{c} \alpha_{i}(t_{k})=\frac{P_{i,k}}{P_{i,\text{rated}}} \end{array}$$ (20)

$$\begin{array}{c} C_{\text{bal,norm}}=\frac{C_{\text{bal}}}{\max(C_{\text{bal}})} \end{array}$$ (21)

where *t* is continuous time in hours (t∈[0,24]); $\alpha_{i}(t_{k})$ is the load rate of feeder *i* at time *t*_*k*_; *P*_*i*,*k*_ is the power of feeder *i* in the time period *t*_*k*_; *P*_*i*,rated_ is the rated capacity of feeder *i*; *n* is *t*he number of feeders for flexible interconnection (generally 2); *K* is the number of *t*ime intervals; *C*_bal_ and *C*_bal,norm_ are the daily average value of load balance difference and the normalized daily average balance degree of the lines interconnected by flexible interconnection devices, respectively.

#### 3.1.2 Daily utilization hours of flexible interconnection devices.

A day (24 hours) is divided into *K* = 96 time periods, with a time interval of 15 minutes. The mutual aid rule requires that the load rate of at least one end of the feeder is greater than the threshold *P*_*t*0_ = 40%, and the mutual aid direction is guaranteed to be from the feeder with a larger load rate to the one with a smaller load rate:


$$\begin{array}{c} P_{\text{mutual},k}=\min\left(P_{n},\frac{\left|\left(P_{a,k}-P_{b,k}\right)\right|}{2},\left(P_{la}-P_{a}\right),\left(P_{lb}-P_{b}\right)\right) \end{array}$$ (22)

$$\begin{array}{c} T_{u}=\frac{1}{P_{n}}\sum_{k=1}^{96}P_{t,k}\cdot \Delta t \end{array}$$ (23)

$$\begin{array}{c} T_{u,\text{norm}}=\frac{T_{u}}{24} \end{array}$$ (24)

where *P*_mutual,*k*_ is the mutual aid power in the *k*-th time period; *P*_*a*,*k*_ and *P*_*b*,*k*_ are the powers of feeders *a* and *b* in the *k*-th time period, respectively; *P*_*n*_ is the rated power of the Flexible Interconnection Device (FID);*P*_*la*_, *P*_*lb*_ represent the rated capacity of feeder a and b; *T*_*u*_ is the daily utilization hours of the FID; and *T*_*u*,norm_ is the normalized daily utilization hours of the FID, Δt=0.25 hours (15-minute interval).

#### 3.1.3 Mutual aid balance strength of flexible interconnection devices.

The positive and negative mutual aid times are counted respectively when the mutual aid conditions are met:


$$\begin{array}{c} C_{p}=\sum_{i=1}^{K}f\left(\alpha_{a}(t_{k})-\alpha_{b}(t_{k})>0\text{ and }(\alpha_{a}(t_{k})>P_{t0}\text{ or }\alpha_{b}(t_{k})>P_{t0})\right) \end{array}$$ (25)

$$\begin{array}{c} C_{n}=\sum_{i=1}^{K}f\left(\alpha_{a}(t_{k})-\alpha_{b}(t_{k})<0\text{ and }(\alpha_{a}(t_{k})>P_{t0}\text{ or }\alpha_{b}(t_{k})>P_{t0})\right) \end{array}$$ (26)

where


$$\begin{array}{c} f(x)=\left\{ \begin{array}{cc} 1 & x\text{ is true}\\ 0 & x\text{ is false} \end{array}\right. \end{array}$$ (27)

$$\begin{array}{c} \text{Eq}=\frac{\min(C_{p},C_{n})}{\max(C_{p},C_{n})+\varepsilon } \end{array}$$ (28)

where *Eq* represents the mutual balancing strength of the flexible interconnection device, *C*_*p*_ and *C*_*n*_ are the positive and negative mutual aid times of the FID, respectively; $\alpha_{a}(t_{k})$ and $\alpha_{b}(t_{k})$ are the load rates of feeders *a* and *b* in the *k*-th time period (before mutual aid); *P*_*t*0_ is the minimum load rate of the feeder for starting power mutual aid; The input of this model mainly includes relevant data of different lines, mainly the maximum load of the area corresponding to the line, which is solved using the maximum load and the relevant typical load rate curve.

In addition, the comparison of characteristic values before and after adding flexible devices is used to prove the necessity of flexible interconnection devices. This study adopts the Inter-Regional Peak-Valley Difference Standard Deviation (IPVSD) to indicate the characteristic value of load fluctuation in each region:


$$\begin{array}{c} \text{IPVSD}=\sqrt{\frac{1}{M}\sum_{i=1}^{M}(PV_{i}-\overset{―}{PV}{)}^{2}} \end{array}$$ (29)

where *M* represents the total number of regions involved in the calculation; *PV*_*i*_ denotes the peak-valley difference of region *i*, and $\overset{―}{PV}=\frac{1}{M}\sum_{i=1}^{M}PV_{i}$ is the mean peak-valley difference across *M* regions.

The mathematical expression for two lines is:


$$\begin{array}{c} 𝐗=\{X_{1}^{1},X_{1}^{2},\ldots ,X_{1}^{N};X_{2}^{1},X_{2}^{2},\ldots ,X_{2}^{N}\} \end{array}$$ (30)

$$\begin{array}{c} 𝐏=\{P_{1}^{1},P_{1}^{2},\ldots ,P_{1}^{N};P_{2}^{1},P_{2}^{2},\ldots ,P_{2}^{N}\} \end{array}$$ (31)

where **X** denotes the spatial coordinates of nodes, which is distinct from the input matrix **X** in Eq. (1) and the solution vector **X** in the CEO algorithm; *N* represents the number of nodes; **X**_1_ represents the first line; **X**_2_ represents the second line; and **P** represents the maximum load of different nodes on the corresponding line. The influence degree of the flexible interconnection device on the two lines can be judged using the indicators calculated above. To ensure the balance of the three indicators, corresponding weight coefficients are set to integrate the indicators into the objective function of the model for the final algorithm optimization:


$$\begin{array}{c} \text{Cost}=\beta_{1}(1-C_{\text{bal,norm}})+\beta_{2}(1-T_{u,\text{norm}})+\beta_{3}(1-\text{Eq})+\beta_{4}(1-C_{\text{norm,comp}})+\beta_{5}\cdot \text{IPVSD} \end{array}$$ (32)

where $\beta_{1},\beta_{2},\beta_{3},\beta_{4},\beta_{5}$ represent the weight coefficients of the corresponding indicators, and Cost represents the final optimization objective.

The objective function Cost is constructed as a weighted penalty function to convert the multi-objective problem into a single-objective one. The weights $\beta_{1}$ to $\beta_{5}$ are determined using the Analytic Hierarchy Process (AHP), prioritizing the reliability (*C*_*bal*_) and economy (*T*_*u*_) of the system. This setup physically represents the total operational “dissatisfaction” of the system, where a lower cost implies a more balanced, economical, and stable grid operation. Cost aggregates normalized deviations from ideal states (1.0 = perfect balance/utilization/complementarity). Minimizing Cost determines a Pareto front over different sets of weight values—a practical single-objective surrogate for multi-objective optimization in engineering deployment.

### 3.2 Model data classification

**Fig 3:**
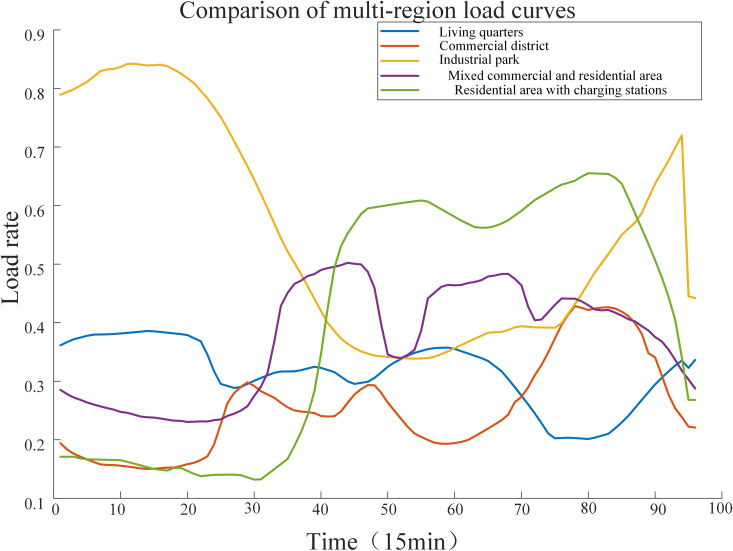
Comparison of actual load rates.

In this paper, load data of different types from existing research results are used for data simulation. The main regional classifications include five types: residential areas, commercial areas, industrial areas, residential-commercial mixed areas, and new communities with electric vehicle charging piles. The typical load curves for different regions are shown in Fig 3. The dataset is generated based on their corresponding load rates; multiplying the load rate by the maximum load of different areas finally obtains the complete load data of different areas, which is used for further analysis and calculation [32].

The training dataset for the hybrid neural network comprises 3000 samples. To enhance model robustness and prevent overfitting caused by pure simulation data, we introduce and augment five real-world load samples obtained from a public utility dataset. These real-world samples capture the stochastic volatility characteristics inherent to actual power grid operation. The forecasting module is validated via a hold-out strategy to ensure its generalization capability. The model input features cover regional areas from 0.2 to 3 km^2^, populations from 5000 to 100000 people, five categories of electricity consumption types, and climate conditions with temperatures ranging from 0 to 40 °C; the model target output is the load curve across 96 time points. To improve the reliability of the overall dataset, this study integrates simulated data with real measured data, as presented in Fig 3.

Additionally, this paper performs data fitting and simulation on loads of different feature types, considering that the electricity load conditions of different regions are different, especially the corresponding peak electricity consumption periods [33].

#### 3.2.1 Residential areas.


$$\begin{array}{c} \gamma_{\text{res}}(t)=0.3+0.2\exp\left(-\frac{(t-7.5{)}^{2}}{2}\right)+0.25\exp\left(-\frac{(t-20{)}^{2}}{3}\right)+N(0,0.02^{2}) \end{array}$$ (33)

It consists of a base value of 0.3 and two Gaussian peaks, centered at 7.5 hours (morning) and 20 hours (evening), respectively, to simulate the morning and evening electricity consumption peaks of residents. In Sections 2.2.1–2.2.5, *t* is continuous time in hours (t∈[0,24]).

#### 3.2.2 Commercial areas.


$$\begin{array}{c} \gamma_{\text{com}}(t)=0.15+0.25\cdot I_{\left[9,17\right]}(t)\cdot (1-0.15\cdot I_{\left[12,13\right]}(t))+N(0,0.03^{2}) \end{array}$$ (34)

where *I*_[*a*,*b*]_(*t*) is an indicator function, which is 1 when t∈[a,b] and 0 otherwise. The base load of 0.15 is superimposed with the active load during working hours (9:00–17:00), and the active load decreases by 15% during the noon period (12:00–13:00). The noise standard deviation increases to 0.03, reflecting the volatility of commercial activities.

#### 3.2.3 Industrial areas.


$$\begin{array}{c} \gamma_{\text{ind}}(t)=0.35+0.05\sin\left(\frac{2\pi t}{24}\right)+N(0,0.02^{2}) \end{array}$$ (35)

A base value of 0.35 is superimposed with a sine fluctuation with an amplitude of 0.05 and a period of 24 hours. The sine term simulates the slight fluctuation of continuous day and night production, and the noise characteristics are the same as those of the residential type.

#### 3.2.4 Residential-commercial mixed areas.


$$\begin{array}{c} \gamma_{\text{mix}}(t)=0.5\alpha_{\text{res}}(t)+0.5\alpha_{\text{com}}(t)+N(0,0.02^{2}) \end{array}$$ (36)

It is the weighted average of residential and commercial curves (50% each), retaining the composite characteristics of residential morning and evening peaks and commercial daytime activities, with the noise level consistent with that of the residential type.

#### 3.2.5 Residential areas with charging piles.


$$\begin{array}{c} \gamma_{\text{res-charg}}(t)=\alpha_{\text{res}}(t)+0.1\exp\left(-\frac{(t-10{)}^{2}}{4}\right)+0.12\exp\left(-\frac{(t-22{)}^{2}}{5}\right)+N(0,0.02^{2}) \end{array}$$ (37)

This simulation data is a variation of the original residential area data, adding the simulated charging load of electric vehicles to the original residential area data.

## 4 Simulation and verification

**Fig 4:**
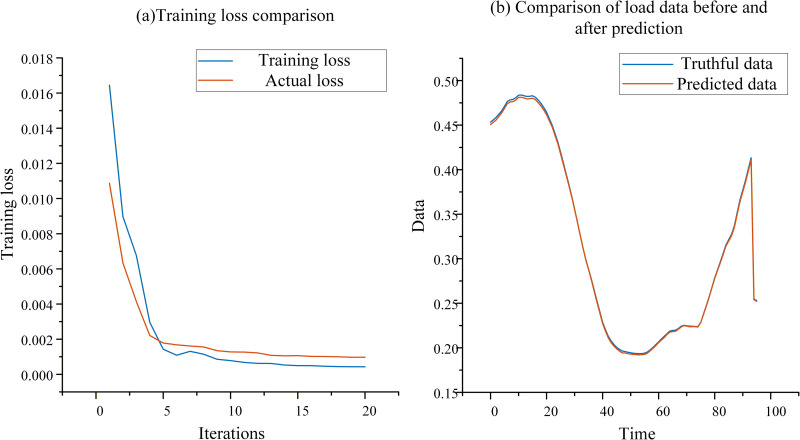
Prediction results of the hybrid neural network.

**Fig 5:**
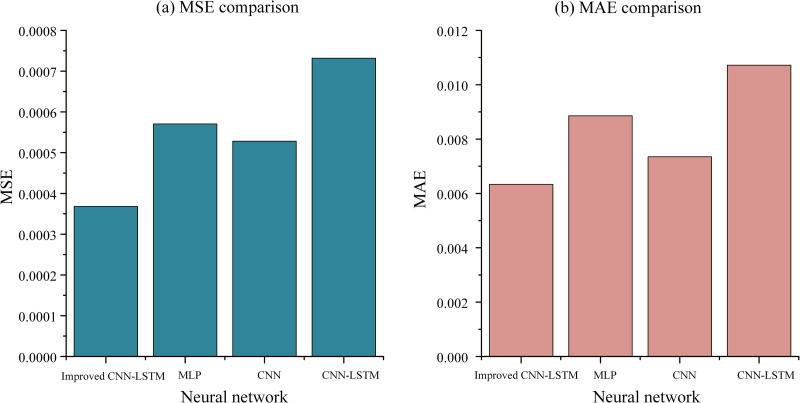
Performance comparison of different neural networks.

The experiment implements the improved CNN-LSTM model using Python and PyTorch, and the optimization algorithm is implemented in MATLAB. The improved CNN-LSTM model captures different spatial patterns through short and long kernel convolutions, uses the BiLSTM module to handle the long-term fluctuation of the load curve, and finally adopts an 8-head attention mechanism to enhance the prediction accuracy of key time points (such as peaks). Finally, the MSE of the improved CNN-LSTM model on the test set is 0.000368, and the MAE is 0.006334, which is superior to the traditional Multi-Layer Perceptron (MLP) (MSE = 0.000571, MAE = 0.008857) and CNN (MSE = 0.000528, MAE = 0.007349) models, verifying its advantages in spatio-temporal feature extraction. The prediction results of the improved CNN-LSTM and the performance comparison of different neural networks are shown in Figs 4 and 5, respectively.

**Fig 6:**
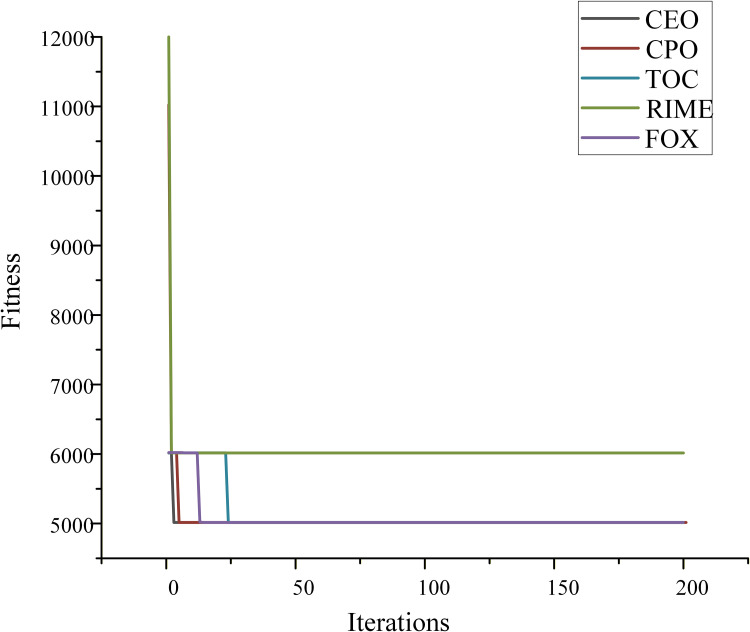
Iteration data comparison of different algorithms.

Using the predicted load curve, the CEO algorithm is used for optimization. The CEO algorithm generates diverse evolutionary directions through hyperchaotic mapping to avoid local optimality, and then adopts a local development mechanism to balance exploration and exploitation. Finally, convergence is achieved after 4 iterations. Compared with other algorithms, the CEO algorithm has improvements in both convergence speed and optimization effect. This paper compares different neural networks using the Crested Porcupine Optimizer (CPO) [34], Tornado Optimizer with Coriolis force (TOC) [35], Rime Optimization Algorithm (RIME) [36], and Fox Optimizer (FOX) [37]. The overall comparison is shown in Fig 6, the iteration data is shown in Table 1, and the performance indicators of the model are shown in Tables 2–5. The optimization process data and final results are provided in S1 File.

**Table 1:** Performance comparison of optimization algorithms.

Algorithm	Objective Function Value	Number of Iterations
CEO	5015.5	4
CPO	5015.7	5
TOC	6015.4	23
RIME	5016.4	63
FOX	5015.5	30

**Table 2:** Optimized results after prediction by improved CNN-LSTM neural network.

Algorithm	*C*_bal,norm_	*T*_*u*,norm_	*E*_*q*_	*C*_norm,comp_	IPVSD_non-inter_	*IPVSD*_inter_
CEO	3.5767	0.53104	0.88939	2.3408	0.08515	0.05924
CPO	3.5733	0.23104	0.57786	2.1452	0.08515	0.07638
TOC	3.5722	0.1428	0.68722	2.2549	0.08515	0.07969
RIME	3.5743	0.35429	0.6	2.0901	0.08515	0.08242
FOX	3.5767	0.43337	0.87879	2.2549	0.08515	0.08278

**Table 3:** Optimized results after prediction by MLP neural network.

Algorithm	*C*_bal,norm_	*T*_*u*,norm_	*E*_*q*_	*C*_norm,comp_	$IPVSD_{non-inter}$	*IPVSD*_inter_
CEO	3.5785	0.56732	0.72518	2.3050	0.07809	0.06294
CPO	3.5698	0.38215	0.71245	2.1987	0.07809	0.07732
TOC	3.5714	0.39841	0.80329	2.1250	0.07809	0.06817
RIME	3.5726	0.52108	0.76542	2.1600	0.07809	0.07751
FOX	3.5779	0.33125	0.69530	2.2800	0.07809	0.07352

**Table 4:** Optimized results after prediction by CNN neural network.

Algorithm	*C*_bal,norm_	*T*_*u*,norm_	*E*_*q*_	*C*_norm,comp_	$IPVSD_{non-inter}$	*IPVSD*_inter_
CEO	3.5738	0.51127	0.79215	2.1400	0.09254	0.07331
CPO	3.5701	0.42653	0.73562	2.2500	0.09254	0.07325
TOC	3.5719	0.35210	0.74890	2.1750	0.09254	0.08732
RIME	3.5719	0.48765	0.76981	2.1550	0.09254	0.07632
FOX	3.5725	0.39876	0.72005	2.2300	0.09254	0.08667

**Table 5:** Optimized results after prediction by CNN-LSTM neural network.

Algorithm	*C*_bal,norm_	*T*_*u*,norm_	*E*_*q*_	*C*_norm,comp_	$IPVSD_{non-inter}$	*IPVSD*_inter_
CEO	3.5723	0.53218	0.81000	2.1300	0.08857	0.06724
CPO	3.5772	0.35109	0.70015	2.2950	0.08857	0.07218
TOC	3.5835	0.57834	0.78520	2.1450	0.08857	0.08565
RIME	3.5807	0.44287	0.74231	2.2100	0.08857	0.08232
FOX	3.5815	0.50219	0.75846	2.1650	0.08857	0.08541

The daily average balance degree of interconnected feeders reflects the load difference between feeders, which is calculated by the spatio-temporal standard deviation of load rates; a larger value indicates better load balance. The daily utilization rate of the device is calculated based on the ratio of mutual aid power to rated power to activate the market share; a larger value indicates more sufficient use of the device. The mutual aid balance strength represents the bidirectional energy flow; a larger value indicates more balanced energy flow. From the comparison between Tables 2 and 3, it can be seen that the normalized indicators of flexible interconnection after CEO optimization using the data predicted by the improved CNN-LSTM neural network are larger, indicating a better flexible interconnection effect.

**Fig 7:**
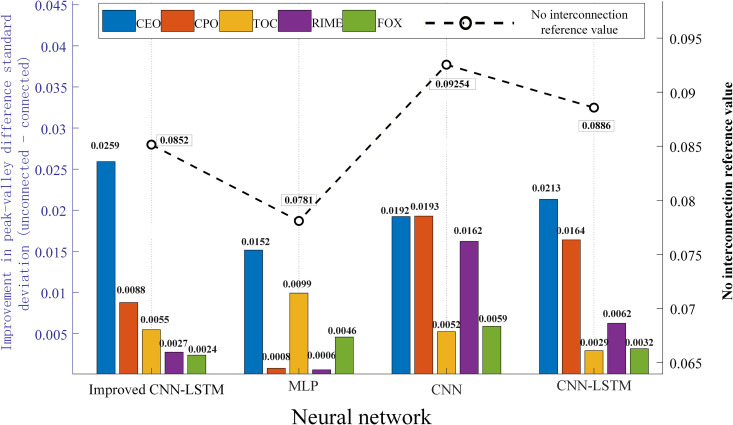
Comparison of final optimization effects.

In addition, the IPVSD in Table 2 decreases from 0.08515 to 0.05924 (a decrease of 30.5%). Fig 7 shows the optimization results using the data predicted by the improved CNN-LSTM neural network, with higher benefits of flexible interconnection. In addition, compared with other optimization methods in Tables 3–5, this optimization method achieves the best effect and the highest reduction rate.

## 5 Conclusion

With the development of new power systems toward intelligence and flexibility, aiming at the key issue of how flexible interconnection technology can improve system reliability and economy, this paper proposes a flexible interconnection planning optimization method based on improved CNN-LSTM prediction and Chaotic Evolutionary Optimization (CEO). The proposed improved CNN-LSTM balances computational efficiency and accuracy, outperforming more complex models like Transformers. As shown in the results, it significantly outperforms standard MLP and CNN baselines. Comparison with the standard CNN-LSTM (Table 5) further validates the efficacy of the proposed multi-scale and attention mechanisms. This method has the advantages of high solution efficiency and strong global optimization capability. By adding flexible interconnection, the inter-regional peak-valley difference in the original power grid is reduced, and the overall operation efficiency of the power grid is improved. In addition, the following conclusions are obtained through theoretical research and simulation experiments:

(1) Aiming at the load forecasting problem, an improved CNN-LSTM network with multi-scale spatio-temporal cross-enhancement is designed, which integrates multi-scale convolution, bi-directional LSTM, and attention mechanism to improve load forecasting accuracy. High-precision prediction at 96 points is realized on the load dataset of 5 types of regions. Simulation results show that this method achieves low Mean Squared Error (MSE = 0.000368) and Mean Absolute Error (MAE = 0.006334) in load forecasting, providing a high-precision data basis for optimization.
(2) A flexible interconnection objective function integrating multiple indicators is constructed, which comprehensively considers multi-dimensional indicators such as balance, reliability, and economy, and realizes efficient optimization through the chaotic optimization algorithm. Simulation results show that the chaotic optimization algorithm can effectively reduce the value of the optimization objective function within a fast number of iterations, and compared with other methods, the reduction is more than 5%, achieving a better optimization effect.
(3) The superior performance of the CEO algorithm can be attributed to the ergodic property of the hyperchaotic map. Unlike random initialization in traditional evolutionary algorithms, chaotic mapping allows the solution to traverse the search space more thoroughly in the early stages, preventing the algorithm from falling into local optima in this high-dimensional non-convex planning problem. Both *C*_bal,norm_ and *T*_*u*,norm_ are better the larger they are. From Tables 2–5, it can be seen that compared to other algorithms, CEO achieves the best optimization based on the optimal neural network, with improvements over other algorithms. *C*_bal,norm_ increases by more than 0.095%, and *T*_*u*,norm_ increases by more than 22%. The method proposed in this paper has good engineering practicality in load forecasting and system optimization. This study adopts Tunable Relative Entropy (TRE) as a complementarity quantification index, among which the CEO algorithm achieves the best indicator, which is at least 3.8% higher than other algorithms, dynamically measuring the spatio-temporal complementarity of load curves between feeders from the perspective of probability distribution. The future work of this study will explore a multi-objective optimization framework, introduce environmental and social benefit indicators, and further improve the practical value of the method.

## Supporting information

S1 File Optimization results. (XLSX)
